# Grb7 SH2 domain structure and interactions with a cyclic peptide inhibitor of cancer cell migration and proliferation

**DOI:** 10.1186/1472-6807-7-58

**Published:** 2007-09-25

**Authors:** Corrine J Porter, Jacqueline M Matthews, Joel P Mackay, Sharon E Pursglove, Jason W Schmidberger, Peter J Leedman, Stephanie C Pero, David N Krag, Matthew CJ Wilce, Jacqueline A Wilce

**Affiliations:** 1 School of Biomedical and Chemical Sciences, University of Western Australia, WA 6009, Australia; 2 Department of Biochemistry and Microbiology, University of Sydney, NSW 2006, Australia; 3 Western Australian Institute of Medical Research, WA 6000, Australia; 4 Department of Surgery and Vermont Cancer Center, University of Vermont, Burlington, VT, USA; 5 Department of Biochemistry and Molecular Biology, Monash University, VIC 3800, Australia

## Abstract

**Background:**

Human growth factor receptor bound protein 7 (Grb7) is an adapter protein that mediates the coupling of tyrosine kinases with their downstream signaling pathways. Grb7 is frequently overexpressed in invasive and metastatic human cancers and is implicated in cancer progression via its interaction with the ErbB2 receptor and focal adhesion kinase (FAK) that play critical roles in cell proliferation and migration. It is thus a prime target for the development of novel anti-cancer therapies. Recently, an inhibitory peptide (G7-18NATE) has been developed which binds specifically to the Grb7 SH2 domain and is able to attenuate cancer cell proliferation and migration in various cancer cell lines.

**Results:**

As a first step towards understanding how Grb7 may be inhibited by G7-18NATE, we solved the crystal structure of the Grb7 SH2 domain to 2.1 Å resolution. We describe the details of the peptide binding site underlying target specificity, as well as the dimer interface of Grb 7 SH2. Dimer formation of Grb7 was determined to be in the μM range using analytical ultracentrifugation for both full-length Grb7 and the SH2 domain alone, suggesting the SH2 domain forms the basis of a physiological dimer. ITC measurements of the interaction of the G7-18NATE peptide with the Grb7 SH2 domain revealed that it binds with a binding affinity of K_d _= ~35.7 μM and NMR spectroscopy titration experiments revealed that peptide binding causes perturbations to both the ligand binding surface of the Grb7 SH2 domain as well as to the dimer interface, suggesting that dimerisation of Grb7 is impacted on by peptide binding.

**Conclusion:**

Together the data allow us to propose a model of the Grb7 SH2 domain/G7-18NATE interaction and to rationalize the basis for the observed binding specificity and affinity. We propose that the current study will assist with the development of second generation Grb7 SH2 domain inhibitors, potentially leading to novel inhibitors of cancer cell migration and invasion.

## Background

Tyrosine kinase signaling pathways play a major role in the regulation of cell growth, division and motility. It is unsurprising, therefore, that aberrations of these pathways can underlie cancerous phenotypes [1,2] and that tyrosine kinase pathways have been the targets of several successful anti-cancer agents [3]. These targets have included extracellular and cytoplasmic domains of receptor tyrosine kinases, but downstream binding partners may also prove to be important targets for new therapeutics [4,5].

Grb7 is a member of a family of adapter proteins that includes Grb10 and Grb14, and serves to couple activated tyrosine kinases to downstream signaling pathways [6]. These proteins share a region with sequence homology to the Mig-10 *C. elegans *gene required for migration of neuronal cells in embryonic development, suggesting a role for the Grb7 family in cell migration [7]. This role is consistent the presence of Grb7 in focal adhesions, where it is bound and phosphorylated by focal adhesion kinase (FAK) in the process of cell migration [8,9]. Grb7 is also found in the cytoplasm where it interacts with other upstream binding partners [10,11] including the members of the ErbB receptor family [12,13] whose activities play a critical role in the regulation of cell proliferation [14,15].

Although the precise downstream activities of Grb7 are not yet known, there is compelling evidence that Grb7 represents an important new cancer target [16]. Grb7 is frequently overexpressed in invasive and metastatic cell lines. Grb7 is tightly co-amplified with the ErbB2 receptor in breast cancer cell lines and there is a strong correlation between ErbB2 and Grb7 over-expression in primary breast cancer specimens [13], as well as in oesophageal and gastric carcinoma [17,18]. Recent data suggest that upregulation of Grb7 impacts on both the proliferative and invasive potential of the cancer cells. An inhibitor peptide specific for Grb7 was shown to inhibit breast cancer cell proliferation with no effect on non-malignant cells [19]. In a separate study, the same Grb7 inhibitor was demonstrated to significantly attenuate the migratory potential of pancreatic cancer cells [20]. Grb7 is thus an important candidate for the development of inhibitors that block aberrant Grb7 downstream signaling in cancer progression.

Members of the Grb7 family share a conserved multi-domain structure comprising an N-terminal proline rich domain, a Ras-associating-like (RA) domain, a plekstrin homology (PH) domain, a C-terminal src-homology 2 (SH2) domain and a region between the PH and SH2 domains termed the BPS domain [21,22]. The interaction with upstream tyrosine kinases is predominantly mediated by the SH2 domain, which recognizes specific phosphotyrosines. The adjacent BPS domain has also been shown to contribute to recognition of the insulin and insulin-like growth factor-1 receptors by Grb7 family members [23,24]. Phosphotyrosine (pY) recognition occurs via a highly positively charged pocket at the surface of the SH2 domain [25,26]. Substrate differentiation is conferred by amino acid residues immediately surrounding the phosphotyrosine and in particular those located C-terminal to the phosphotyrosine [27].

Despite the SH2 domains of Grb7, Grb10 and Grb14 proteins exhibiting between 62 – 72% sequence identity, the domains recognise different phosphotyrosine containing sequences. The Grb7 SH2 domain displays a strong preference for phosphotyrosines contained within a pYXN motif, where X is any amino acid [28]. The presence of the asparagine at the +2 position (relative to pY) is thought to facilitate the formation of a turn in this region, enhancing the interaction with the Grb7 SH2 domain [29]. In contrast, Grb14 SH2 domain recognition sequences contain a hydrophobic residue at the +3 position relative to the phosphotyrosine [30,31]. A consensus binding sequence for the Grb10 SH2 domain has yet to be determined although several pY binding sites have been identified, including Tyr-771 of PDGF-Rβ, Tyr-929 of ELK and the phosphorylated activation loop of the Insulin Receptor [22,32-35].

Due to the implication of Grb7 in cancer cell progression, an inhibitor specific for Grb7 SH2 domain has been developed that targets the Grb7 SH2 pY peptide binding site [28]. Such a peptide provides the opportunity to test the role of Grb7 in cancer progression, and may potentially lead to novel anti-cancer therapeutics [16]. The peptide, termed G7-18NATE (Grb7-peptide18-No Arms Thioether), was developed using phage display screening for peptides able to specifically bind Grb7 SH2 [28]. G7-18NATE bound to the Grb7 SH2 domain, but not to Grb14 SH2 domain or to the Grb2 SH2 domain that has also been shown to preferentially bind to a YXN motif. Furthermore, in a recent study by Tanaka and colleagues, G7-18NATE was shown to not only selectively block the interaction between Grb7 and FAK *in vivo*, but to significantly attenuate the migration of pancreatic cancer cells [20]. In addition to effects on cell migration, cell permeable G7-18NATE peptides have recently been shown by Pero et. al. to inhibit the proliferation in a variety of different breast cancer cells with no effect on non-malignant cells. Co-treatment of cell permeable G7-18NATE peptide plus chemotherapeutic drugs Doxorubicin or Herceptin resulted in significant reduction of the drug EC50 value in breast cancer cells [19]. G7-18NATE thus represents the first specific inhibitor of Grb7 and the first stage in the development of a potential anti-cancer therapeutic. This Grb7 inhibitory peptide has potential to be developed as a therapeutic agent alone, in combination with traditional chemotherapy, or in combination with other targeting molecules for treatment of cancer.

As a step towards determining the means by which Grb7 SH2 recognizes its specific pY peptide substrates and the G7-18NATE peptide inhibitor we report the crystal structure of the human Grb7 SH2 domain to 2.1 Å resolution. This facilitates the first high resolution comparison of the Grb7 SH2 domain with Grb10 and Grb 14 SH2 domains, allowing us to better understand the basis for their differential binding specificities [36,37]. We also describe the structural basis for the previously reported Grb7 SH2 domain dimerisation [38,39] and show this also takes place for full-length Grb7, with implications for the regulatory mechanism of Grb7 activity. Furthermore, we present biophysical studies of the interaction between the G7-18NATE peptide and the Grb7 SH2 domain, using isothermal titration calorimetry and NMR spectroscopy, which provide insight into the basis of their binding affinity and specificity. Together, the data has allowed us to propose a model of the interaction that may assist the further development of potent and specific inhibitors of Grb7.

## Results

### The overall structure of the Grb7 SH2 domain

As a first step towards describing the G7-18NATE/Grb7 interaction we solved the crystal structure of the Grb7 SH2 domain to 2.1 Å. This was achieved using molecular replacement, employing the crystal structure of the Grb10γ SH2 as a search model (PDB:1NRV [36]). The final Grb7 SH2 model consists of four protein chains each containing residues 420–532 of the human Grb7 protein, 181 water molecules and 7 sulphate anions, and has an R-factor of 20.1% (R_free _= 25.5 %) (Table 1). Interpretable electron density was missing for the 7 N-terminal residues of the construct (GSPASGT) in all four chains. The side-chains of several residues in the BC, DE and CD loops and the αA helix were also omitted from the final model due to poorly defined electron density. The pairwise α-carbon root mean square deviations (r.m.s.d.) for the four protomers are between 0.23 and 0.95 Å.

**Table 1:** Grb7 SH2 domain Data Collection and Refinement Statistics

**Grb7 SH2**	
Data Collection	
Space group	*P2*_*1*_*2*_*1*_*2*_*1*_
Unit Cell (Å)	*a *= 62.6
	*b = *63.9
	*c = *105.7
	α *= β = γ = *90°
Molecules in the ASU	4
Resolution range (Å)^a^	44 – 2.1 (2.18 – 2.1)
Observations	79384
Unique reflections	25401
Completeness (%)^a^	99.1 (99.4)
I/σ^a^	12.6 (2.2)
R_merge _(%)^a, b^	8.4 (50.5)
Wilson B (Å)^a^	28.4
	
Refinement	
Resolution limits (Å)^a^	30 – 2.10 (2.15 – 2.10)
No. of reflections^a^	23949 (1731)
R_cryst _(%)^a^	20.1 (25.4)
R_free _(%)^a, c^	25.5 (28.9)
Protein atoms	3733
Water molecules	181
Other molecules	7 SO_4_^2-^
r.m.s.d. from ideal values ideaiations	
Bond lengths (Å)	0.014
Bond angles (°)	1.486
Average B-factors (Å)^3^	
Protein	38.1
Water	40.6
Ions	58.7

^a^Values in parentheses are for the highest resolution shell happiness

^b^*R*_merge _= Σ |I – < I >|/Σ I where I is the observed diffraction intensity of the jth observation of reflection hkl and < I > is the average diffraction intensity of all measurements of reflection hkl.

^c^R_free _= Σ | |F_o_|-|F_c_|/Σ |F_o_| where |F_o_| and |F_c_| are the observed and calculated factors respectively.

**Figure 1:**
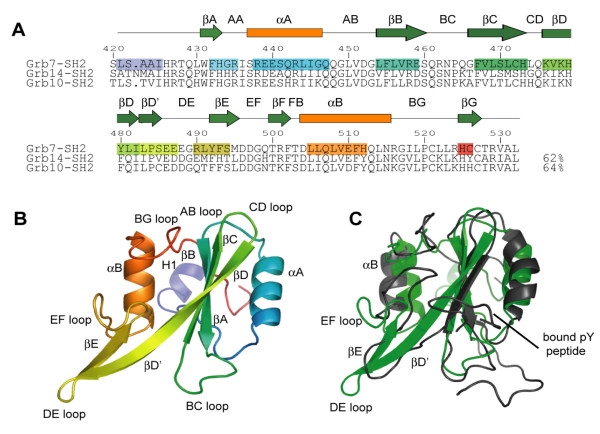
**Crystal structure of the Grb7 SH2 domain**. (a) Structure based sequence alignment of Grb7 SH2, Grb14 SH2 (2AUG; [37]) and Grb10 SH2 (1NRV; [36]). Secondary structure elements present in the Grb7 SH2 structure as determined by WHATIF [71] are shaded from purple at the N-terminus to red at the C-terminus. Secondary structure elements of the canonical SH2 domain as defined by Eck *et al*. [41] are shown in green and orange symbols above the sequences. The boundaries of these elements differ slightly from that observed in the Grb7 SH2 domain. Residue number is for the Grb7 SH2 domain (b) Cartoon representation of the Grb7 SH2 domain shaded from purple at the N-terminus to red at the C-terminus. The extended DE loop distinguishes this family of SH2 domains from others. (c) A structural comparison of the Grb7 SH2 domain (green) with the Grb7 SH2 domain bound to an ErbB2 derived phosphopeptide (1MW4; black; [29]). The location of the bound phosphopeptide is indicated.

The monomer fold of the Grb7 SH2 domain is typical of the SH2 domain family, forming a βαβββββαβ configuration [40] (Figure 1). The standard nomenclature for SH2 domain secondary structures devised by Eck *et al*. [41]is adopted throughout this report. Thus the Grb7 SH2 domain comprises a central antiparallel β-sheet, formed by the βB, βC and βD strands, which is sandwiched by the two α-helices, αA and αB. An extension of the βD strand (termed βD'), along with the βE strand, forms a second, smaller anti-parallel β-sheet. An N-terminal helix was observed prior to the start of the SH2 domain and designated the name H1. The βF strand, observed in other SH2 domains, does not form in Grb7.

### Comparison with other SH2 domains and the Grb7 SH2 NMR derived structure

The Grb7 SH2 domain superposes with Grb14 (PDB:2AUG; [37]) and Grb 10 (PDB:1NRV; [36]) SH2 domains, across Grb7 SH2 residues 431–523, with α-carbon r.m.s.d.s of 1.24 Å and 0.88 Å respectively. Relative to the archetypal Src SH2 domain (1SPS; [42]) the Grb7 SH2 domain shows a slightly greater divergence due to loop structure differences (α-carbon r.m.s.d. of 1.65 Å). This is, in part, due to a three residue insertion in the acidic DE loop characteristic of the Grb7 family members. The CD loop is also shorter by five residues. This combination of differences is, to the best of our knowledge, unique to Grb7 family SH2 domains.

The Grb7 SH2 structure differs markedly from that in complex with the ErbB2 receptor derived peptide, pY1139 determined using NMR spectroscopy (1MW4, representative model 5; [29]) with the two structures overlaying for residues 431–523 with an α-carbon r.m.s.d. value of 3.9 Å (Figure 1C). The NMR derived Grb7 SH2 structure appears to be loosely defined by the NMR derived constraints relative to other SH2 domain structures. More importantly, however, the NMR structure deviates from the crystallographic Grb7 SH2 domain in the region of the DE loop which is not extended in the NMR structure, due to an alternative hydrogen bonding network between the βD' and βE sheets. This difference propagates to a rearrangement of the EF loop, βB helix and BG loop that follow. Whilst it may be argued that these differences reflect structural changes that occur upon pY peptide binding, such dramatic structural rearrangements, that involve a rearrangement of a hydrogen bond network, have not previously been observed upon peptide binding to SH2 domains, although subtler differences have [43-45]. Owing to the considerable differences between the NMR derived Grb7 SH2 domain and all other SH2 domain structures (ligand bound and unbound), we propose that the current crystallographically derived structure is a more accurate model of the Grb7 SH2 domain, and a better starting point for modeling interactions with bound ligands.

### The ligand binding site assumes a bound BC loop conformation

**Figure 2:**
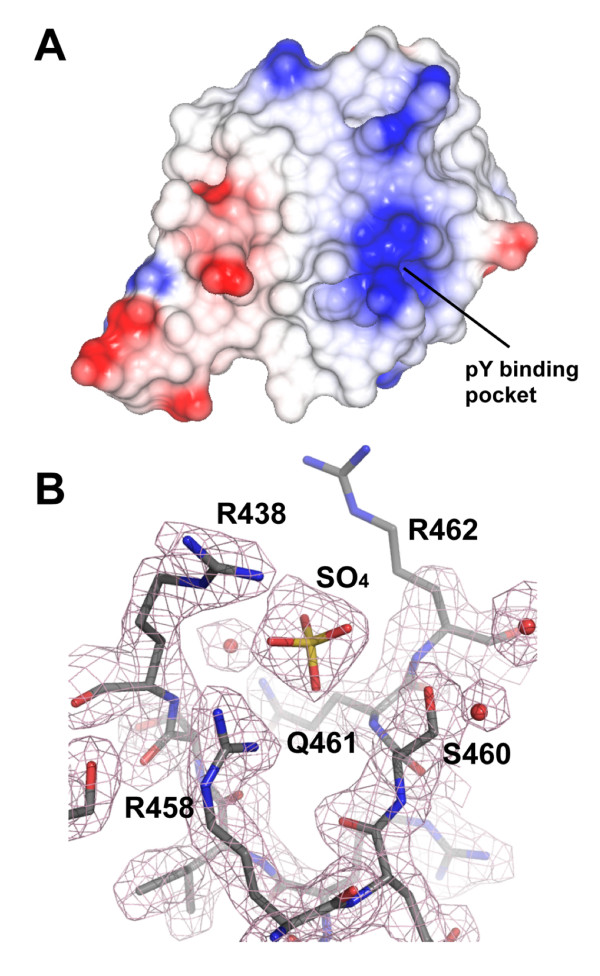
**The phosphate binding pocket of the Grb7 SH2 domain**. (a) Electrostatic potential surface of Grb7 SH2 generated using CCP4MG [80]. Positively charged electrostatic potential is coloured blue and negatively charged electrostatic potential is coloured red. The positions of the phosphate binding pocket is indicated. (b) A 2F_o _- F_c _electron density map depicting the phosphate binding pocket of Grb7 SH2. A sulphate ion co-crystallised in this pocket in all four molecules in the asymmetric unit. The map is contoured at 1 σ. R438, R458, Q461 and S460 form direct contacts with the sulphate ion and are labeled. The side-chain of R462 lacks well defined density and is probably fairly mobile in the crystal.

The ligand binding site within SH2 domains comprises the phosphotyrosine (pY) binding pocket and adjacent peptide binding site that confers specificity for the residues immediately C-terminal to the pY [40]. The Grb7 SH2 pY binding pocket exists in a cleft formed by residues in the central β-sheet, the αA and the BC loop (also referred to as the phosphate binding loop) and possesses a large positive potential for interacting with the phosphotyrosine (Figure 2A). Although the BC loop within *apo*-SH2 domain structures usually exhibits poorly defined electron density [25], the Grb7 SH2 domain BC loop is well defined and closely resembles that of SH2 domains in their peptide bound conformation. The BC loop is held in this conformation by a complex network of hydrogen bond contacts formed with a sulphate anion that appears to mimic the phosphate moiety of the natural ligand (Figure 2B). These contacts include a bidentate ionic interaction with the side-chain of the invariant Arg 458 (βB5). Additional hydrogen bonds are formed with the side-chains of Arg 438 (αA2), Ser 460 (βB7) and the main-chain amide of Gln 461 (BC1).

The adjacent peptide binding site of the Grb7 SH2 domain differs from that observed in typical SH2 domains, such as the archetypal Src SH2 domain, which bind phosphopeptides in an extended conformation [25]. In these SH2 domains, the side-chain of the +3 residue of the phosphopeptide is accommodated within a hydrophobic pocket that is lined by residues in the BG and EF loops, along with the βD strand and αB helix [26]. Analysis of the molecular surface of the Grb7 SH2 domain indicates that it lacks this classical +3 binding pocket. Instead, it is filled by the side-chain of the BG3 residue (Ile 518 which forms a Van der Waals contact with the α-carbon of the EF1 residue (Asp 496) sealing off the pocket. This is consistent with the Grb7 SH2 domain binding a peptide with a turn-containing structure as displayed by the Grb2 and Gads SH2 domains [46,47].

### The Grb7 SH2 dimer interface

**Figure 3:**
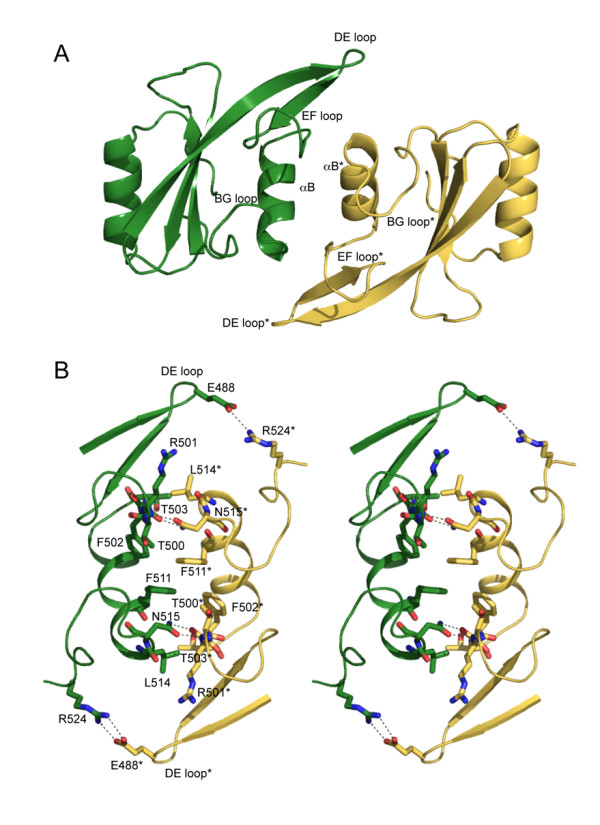
**A stereoview of the Grb7 SH2 domain dimersiation**. (a) Cartoon representation of the Grb7 SH2 domain dimer. The secondary structure elements that form the dimerisation interface are labeled. (b) The Grb7 SH2 domain dimer interface. Residues that form the dimerisation interface are shown. Hydrogen bonds are shown as broken lines. The interaction between R524 and E488 is absent from the AB dimer interface. In both panels the D chain is coloured yellow and indicated by a * and the C chain is coloured green.

The Grb7 SH2 domain crystallised as a dimer, forming a dimerisation interface (between chains A and B and between chains C and D in the asymmetric unit) analogous to that observed for Grb10 and Grb14 domain structures [36,37]. The Grb7 SH2 dimer is formed by the anti-parallel arrangement of two SH2 domains with residues in the βE strand, EF to FB loop region, αB helix and BG loop contributing to the dimer interface (Figure 3). Phe 511 (αB8), the mutation of which has previously been shown to abrogate Grb7 SH2 dimerisation [38], occupies a key position at the centre of the dimerisation interface forming hydrophobic contacts with its equivalent in the opposing protomer (Phe 511*; note that residues in the second protomer are designated by *) and the side-chains of Thr 500*, Phe 502, and Asn 515 (αB12). The side-chains of Thr 503 (FB1) and Leu* 514 (αB11) pack together and this interaction caps the dimerisation interface. Dimer formation is also stabilised by a number of hydrogen bonds. Both OD1 and ND1 of Asn 515 (αB12) form hydrogen bond contacts to the main chain atoms of Arg 501*. The side-chain of Gln 507 (αB4) is hydrogen bonded to the backbone carbonyl of Glu 510* (αB7*). The side-chain of Glu 488 (DE3) was also found to form two hydrogen bonds to the side-chain of Arg 524* (BG9*) in one of the two dimers. A similar network of hydrophobic and hydrophilic interactions stabilises the Grb10 SH2 dimer [36]. The loss of accessible surface area upon dimerisation is about 500 Å^2 ^per monomer, representing approximately 7 % of the monomer surface area. The intermolecular interactions between the two protomers that make up the dimer may help define the positions of residues in the BG and EF loops. This is reflected in the B-factors of these residues (amino acids 496 – 499 and 516 – 524), which are lower than the B-factors determined for residues in other loop regions.

A second dimerisation interface (between chains A and D) was also observed within the asymmetric unit. This interface is not formed within the crystal packing of other Grb7 family member SH2 domains. It occurs with a buried surface area of approximately 400 Å^2 ^per monomer and involves contacts between the AA and BC loops and the αA helix of the A chain, and the βD, βD' and βE strands and the BC, DE and EF loops of the D chain. This buried surface area is smaller than that between the AB and CD dimers and is peculiar to this Grb7 SH2 domain crystal form. Since this interface does not involve Phe 511, shown to be critical to dimerisation in solution, we propose that this is not the dimer that predominates in solution.

### Full-length Grb7 dimerises *in vitro *via its SH2 domain

**Figure 4:**
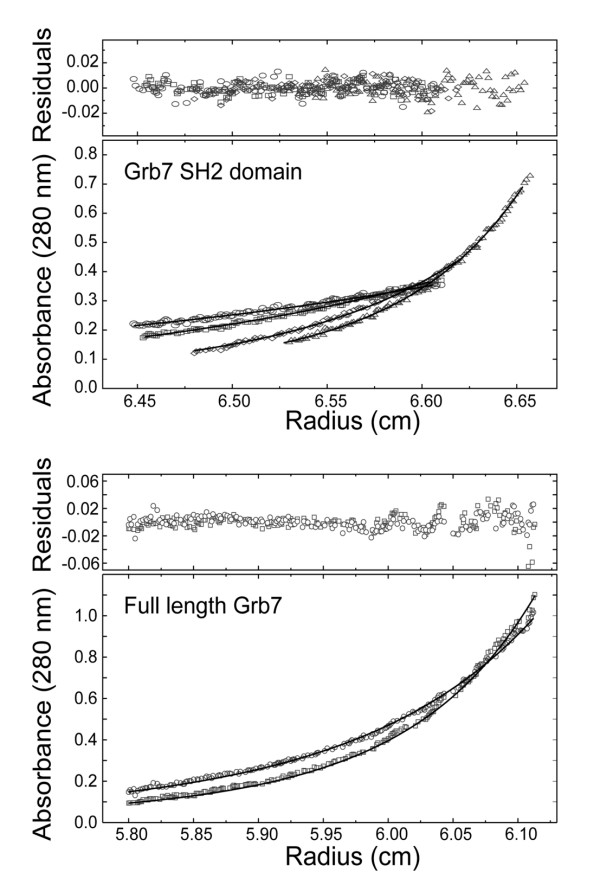
**Sedimentation equilibrium analysis of the Grb7 SH2 and Grb7**. (a) Absorbance at 280 nm verses radius data at sedimentation equilibrium for Grb7 SH2 at an initial loading concentration of 36 μM. The data collected at 14,000 rpm (*circles*), 16,600 rpm (*squares*), 24,300 rpm (*diamonds*) and 28,800 rpm (*triangles*) were fitted simultaneously using the nonlinear regression program [48]. (b) Absorbance at 280 nm verses radius data at sedimentation equilibrium for Grb7 at an initial loading concentrations of 12 μM. The data collected at 10,000 rpm (*circles*) and 11,800 rpm (*squares*) were fitted simultaneously using the nonlinear regression program NONLIN [77]. The *solid line *represents the calculated fit to a monomer-dimer model. The residuals of the fit are shown in the *upper panels*. Samples were in 50 mM MES pH 6.6, 100 mM NaCl and 1 mM DTT. The experiments were conducted at 20°C.

The observation of dimer formation in the crystal structure prompted us to investigate the strength of the interaction between Grb7 SH2 domains as well as between full-length Grb7 molecules. The dimer dissociation constant was calculated for both the Grb7 SH2 domain and full-length Grb7 at 20°C using analytical ultracentrifugation. Figure 4 shows profiles recorded for the two proteins that could be fitted globally to a monomer-dimer self-association model using NONLIN [48,49]. This yielded a dissociation equilibrium constant for the Grb7 SH2 domain of 21.8 μM. This represents a slightly weaker association than that determined in previously reported experiments conducted at 4°C [38], consistent with the lesser role of hydrophobic interactions at the higher temperature. The profiles obtained for full-length Grb7 at 12 μM yielded a dissociation equilibrium constant of 11 μM. Although some sample heterogeneity, possibly due to protein aggregation or degradation, prevented a simultaneous fit with profiles recorded at higher concentrations, the data demonstrated that full-length Grb7 forms dimers in vitro with a similar affinity to that exhibited by the SH2 domain alone. This suggests that the Grb7 molecule may exist as a dimer *in vivo *via its SH2 domain, as also thought to occur for the Grb10 and Grb14 molecules [36,37].

### The G7-18NATE peptide binds Grb7 SH2 with micromolar affinity

**Figure 5:**
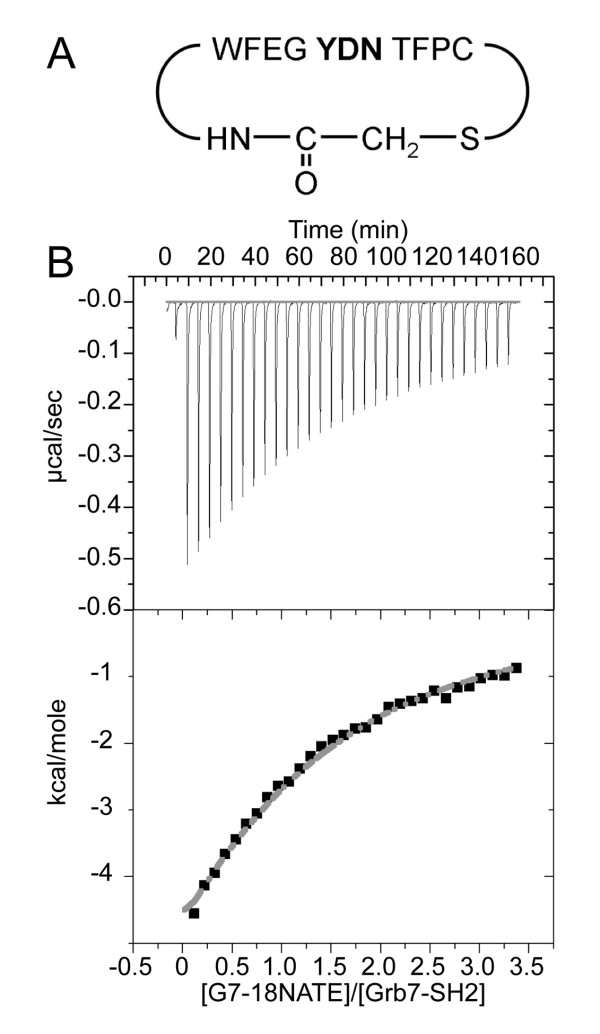
**Isothermal titration calorimetric measurement of G7-18NATE peptide binding to the Grb7 SH2 domain **(a) The sequence of the G7-18NATE peptide used in this study. (b) ITC data showing the titration of G7-18NATE into Grb7 SH2. The samples were prepared in 50 mM MES pH 6.6, 100 mM NaCl and 1 mM DTT. ITC experiments were carried out in duplicate at 25°C with Grb7 SH2 in the cell at 25 μM and G7-18NATE in the syringe at 440 μM. The upper panel shows the raw data while the lower panel shows the data after peak integration and subtraction of the heat-of-dilution control. In this panel the grey line represents the fit to a 1:1 binding model.

The G7-18NATE peptide (Figure 5A) is the first non-phosphorylated peptide to be discovered with binding specificity for the Grb7 SH2 domain. It has been found to bind to the Grb7 SH2 domain, preferentially over the Grb14 SH2 domain, and have the capacity to interfere with Grb7 interactions with the ErbB family of receptors [28] and FAK *in vivo *[20]. These interactions may underlie the observed inhibition of breast cancer cell proliferation and decrease in cell migration of pancreatic cancer cells treated with G7-18NATE, making it of considerable importance to further characterise this peptide and its interactions with Grb7 SH2 domain. Isothermal titration calorimetry was thus used to determine the binding affinity of G7-18NATE for the Grb7 SH2 domain (Figure 5B). This data showed that the peptide bound to the SH2 domain with an affinity of K_d _= ~35.7 μ M. This is only an order of magnitude less than the K_d _= 2.3 μM measured by Lyons and co-workers for the binding of the Grb7 SH2 domain to pY1139, a native phosphopeptide ligand derived from the ErbB2 receptor [39].

### The G7-18NATE peptide perturbs Grb7 SH2 residues at the peptide binding site and the dimerisation interface

**Figure 6:**
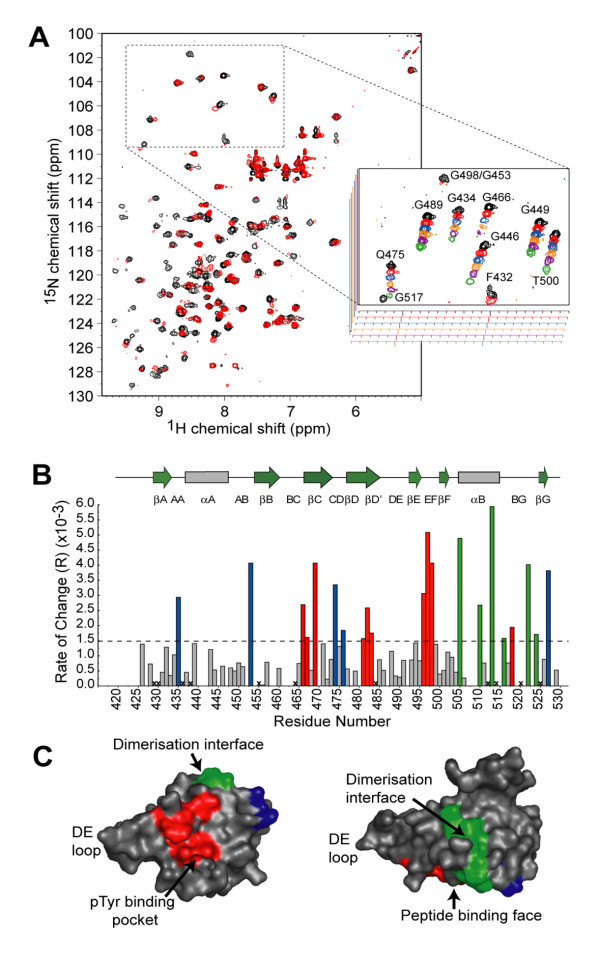
**G7-18NATE binding surface on the Grb7 SH2 domain**. (a) An overlay of the ^15^N, ^1^H-HSQC spectra of Grb7 SH2 alone (black) and in the presence of two molar equivalents of G7-18NATE (red). The insert shows changes to signals in the boxed region of the HSQC spectrum over the course of the titration. The ratio of G7-18NATE to Grb7 SH2 is 0:1, 1:4, 1:2, 3:4, 1:1 and 2:1 in the black, red, blue, orange, purple and green spectra respectively. (b) The rate of change of peak volume over the titration series versus Grb7 SH2 domain residue number. The broken line indicates the mean rate of change. Residues that exhibited rates of change greater than this value are coloured according to their location in the Grb7 SH2 domain structure. (c) Surface representation of the Grb7 SH2 domain with residues affected by G7-18NATE binding coloured. The majority of residues cluster onto either the phosphopeptide binding surface (red) or the dimerisation interface (green). Residues shown in blue were also affected by G7-18NATE binding but do not cluster on one surface. The two images are related by a 90° rotation about the horizontal axis of the page.

In order to further characterize the Grb7 SH2 domain/G7-18NATE interaction NMR titration experiments were used to identify sites of Grb7 SH2 perturbation upon binding by the G7-18NATE peptide. ^1^H-^15^N-HSQC spectra were acquired for ^15^N-labelled Grb7 SH2 as G7-18NATE was titrated in to slight excess (Figure 6A). Approximately 80% of the amide crosspeaks exhibited line broadening, consistent with an 'intermediate' rate of exchange of free and bound forms of the protein (where the exchange rate is close to the chemical shift differences of the resonances between free and bound forms). Other peaks remained unperturbed throughout the titration or exhibited a chemical shift change rather than linebroadening. This behavior is common for interactions for which the dissociation constant is between 10^-3 ^and 10^-6 ^M.

This titration experiment revealed which residues of Grb7 SH2 domain are perturbed upon G7-18NATE binding. Grb7 SH2 domain amide resonances have previously been assigned and were kindly provided [50]. A plot of the rate of change of signal intensity for each amino acid (as a function of added peptide concentration) is shown in Figure 6B. Those that broadened most rapidly (above the mean value of R = 1.5 × 10^-3^) were considered significant. Upon mapping these residues onto the structure of the Grb7 SH2 domain, they clustered into two regions on the protein surface (Figure 6C). The first region (red) is on the peptide binding face of the domain, and includes residues from the βC and βDβD' sheets as well as the EF loop. These perturbations are similar in position to those reported for the pY1139 peptide binding to Grb7 SH2 [50] and are consistent with the same binding site being adopted by G7-18NATE.

The second region (green) occurs along the αB helix and BG loop, which forms part of the dimerisation interface of Grb7 SH2, where direct contacts with the peptide ligand would not be expected to take place. Such perturbations may result from impact of G7-18NATE binding on the Grb7 SH2 domain dimer-monomer equilibrium. A shift from dimeric to monomeric Grb7 SH2 domain has previously been reported upon binding by the pY1139 peptide [50]. This was verified by NMR relaxation time measurements, which reduced from 21.5 ns to 11.7 ns upon pY1139 binding [39]. The perturbations at the Grb7 SH2 domain dimerisation interface upon G7-18NATE binding are thus also likely to be due to a shift in equilibrium towards the monomer.

Other Grb7 SH2 residues perturbed by G7-18NATE binding were located outside these two domains and may result from distal structural rearrangements in these regions resulting from the peptide binding. Interestingly, no major perturbation of the Grb7 SH2 DE loop region was observed in either our study of G7-18NATE, or the study of pY1139 binding. It is thus unlikely that the conformational rearrangement of the DE loop reported in the NMR derived structure of the Grb7 SH2/pY1139 peptide complex occurs upon to peptide binding.

### Model of the G7-18NATE/Grb7 SH2 complex

**Figure 7:**
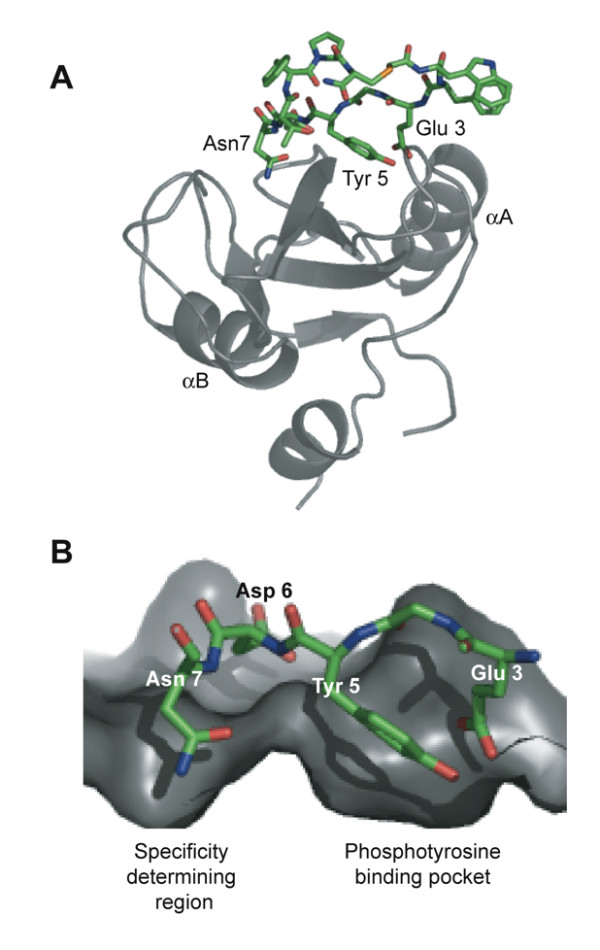
**Model of the Grb7 SH2/G7-18NATE complex**. (a) The modeled structure of the complex. The Grb7 SH2 domain is shown in grey in cartoon representation and G7-18NATE in stick representation with carbon atoms coloured green. The positions of the two α-helices are indicated. The model was prepared using the structure of the Grb2 SH2:pYVN complex (1JYR; [52]) as a template and energy minimised using NAMD [79]. (b) A close up of the Grb7 SH2/G718-NATE interface. The Glu3 and Tyr5 side-chains occupy the phosphate binding pocket while the Asn7 side-chain occupies a second pocket in the specificity determining region of the domain. Only Glu3, Tyr5, Asp6 and Asn7 are shown for clarity.

Together the structural data has allowed a model of the G7-18NATE peptide interacting with the Grb7 SH2 domain to be constructed. In separate studies we have shown that the G7-18NATE peptide has an intrinsic propensity for a turn conformation about the YDN motif [51]. The current study also confirmed that G7-18NATE binding impacts on the Grb7 SH2 domain surface in a similar way to the pY1139 peptide, and is thus likely to bind at the classical peptide binding site. A model of the G7-18NATE peptide was hence positioned at the peptide binding site of the crystallographically determined Grb7 SH2 domain structure by analogy to the Shc derived peptide (PSpYVNVQN) which interacts with Grb 2 SH2 domain in a turn conformation (1JYR [52]) (Figure 7). In this model Asn7, in the +2 position relative to Tyr5, is positioned to form hydrogen bond interactions via its sidechain carbonyl and amine functionalities with backbone NH and carbonyl groups of the βD6 residue. The non-phosphorylated tyrosine (Tyr5) is able to adopt a position in which its hydroxyl is positioned in the positively charged pY binding site. The aromatic ring of Tyr 5 forms Van der Waals contacts with the βD6 residue sidechain (Leu 481) that may be important for the positioning of the peptide ligand. Glu3 is also positioned in the pY binding site. Electrostatic interactions formed by the Glu3 sidechain may compensate for the lack of a phosphate moiety on the tyrosine. The side chain of Asp6 is oriented away from the binding cleft and is in proximity of the backbone NH of the βD8 (His483) residue with which it could form a hydrogen bond. Other residues, constrained in a cyclical conformation, may make contacts at the surface of βC, βD' and EF loop residues shown to be perturbed by NMR spectroscopy. Whilst this model should remain speculative, it suggests a likely basis for the relatively high affinity and specific interaction between G7-18NATE and the Grb7 SH2 domain.

## Discussion

The Grb7 SH2 domain has been identified as a potential target for the development of agents to reduce the invasive potential of cancer cells in which Grb7 is overexpressed. Its crystal structure to 2.1 Å resolution provides structural detail for understanding the basis of interaction with natural targets and designed inhibitors. We have shown that the Grb7 SH2 domain adopts the classic SH2 domain fold excepting, as also observed for Grb10 and Grb14 SH2 domains, an extension of the βD' and βE strands resulting in a longer DE loop [36,37,42]. Along with residues in the EF loop and αB helix, the extended DE loop contributes to the dimerisation Grb7 SH2 domain. Also like the Grb10 and Grb14 SH2 domains, the Grb7 SH2 domain +3 pY peptide binding site is occluded, suggesting that peptides are preferentially bound in a turn-like conformation [36,37].

Despite the similarity of the Grb7/10/14 SH2 domains only Grb7 shows preferential binding for a YXN motif [6,28,53]. Grb7 SH2 is reported to interact strongly with Tyr-1139 (YVNQ) of the ErbB2 receptor while Grb14 does not [54,55]. Furthermore, the G7-18NATE peptide, selected on the basis of its YXN motif, was shown to bind to the Grb7 SH2 domain but not to the Grb14 SH2 domain [28]. A key mutagenesis study investigating the molecular basis for ErbB2 receptor binding by Grb7 revealed the critical role of the βD6 residue (Leu 481) in Grb7 SH2 domain target recognition [55]. When this residue was mutated to the Grb14 equivalent residue (Leu - > Gln), binding to the ErbB2 receptor was completely abrogated. Conversely, the mutation of the βD6 residue of Grb14 SH2 domain to the Grb7 equivalent residue (Gln - > Leu) imparted it with high affinity for ErbB2. The Leu 481 residue thus holds the key to Grb7 SH2 domain binding specificity.

The current structural data allows the impact of the βD6 residue on the structure of Grb7 SH2 domain to be examined. The βD6 residue occurs at the peptide binding interface at the "specificity determining region" alongside the phosphotyrosine binding pocket. By analogy to the interaction between the Grb2 SH2 domain and pYXN containing phosphopeptides, the βD6 residue forms critical hydrogen bond contacts with the side-chain functionalities of the +2 Asn via both its backbone NH and carbonyl moieties [46,52,56]. This is consistent with the perturbation of residue 481 observed upon the titration of G7-18NATE as detected in the current NMR experiment. Interestingly, a direct structural comparison of the crystallographically determined Grb7 and Grb14 SH2 domains shows that the βD6 residue backbone atoms are equivalently positioned in Grb7 and Grb14. Thus no direct disturbance to the formation of hydrogen bonds to the +2 Asn would be expected to occur when the βD6 residue is a Gln rather than a Leu. Instead, Grb7 binding to a YXN motif is likely to be impacted on by the position and chemical nature of the βD6 residue sidechain. The sidechain of βD6 residue forms part of the phosphotyrosine binding pocket and it may be that subtle changes to the phosphotyrosine binding position, caused by mutation of the βD6 residue from a Leu to a Gln, results in the observed loss of affinity for a YXN motif.

The Grb7 SH2 domain was found to be dimeric in the crystal structure and in solution, forming an analogous dimerisation interface to that observed for Grb10 and Grb14 SH2 domains [36-39]. Dimerisation of full-length Grb7 was demonstrated to occur in solution with a K_d _of the same order of magnitude as that obtained for the SH2 domain alone. This suggests that the Grb7 SH2 domain forms the principle dimerisation interface within the Grb7 dimer. A similar conclusion was reached for Grb10 by Hubbard and co-workers who found that mutation of Phe 515 to an Arg in full-length Grb10γ (the mutation which produced monomeric Grb10 SH2 domain) generates a monomeric form of the full-length protein [36].

The physiological importance of Grb7 SH2 domain dimerisation is still uncertain. It has been suggested for Grb10 and Grb14, that the dimeric state of the protein may contribute to a cooperative association of two Grb molecules to form a dimeric activated tyrosine kinase receptor [36,37]. More recently it has been shown that Grb14 binding to the insulin receptor is enhanced by its dimerisation [37]. In this and other studies, however, it has been shown that peptide binding by the Grb7 SH2 domain impacts on the dimer interface and shifts the dimer-monomer equilibrium towards the monomer [39,50]. Whether Grb14 and Grb10 also undergo a dimer to monomer transition upon peptide binding is yet to be ascertained, but this would seem to be in conflict with enhanced tyrosine kinase receptor binding by dimer formation. It may be that a dimer to monomer transition upon peptide binding is peculiar to Grb7.

The structure of the Grb7 SH2 domain, elucidated by our crystallographic study, has permitted its interaction with a recently developed inhibitor peptide to be examined more closely. The G7-18NATE peptide is an important lead peptide in the development of novel therapeutics targeting cancer cell migration [20,28]. We report a binding affinity of K_d _= ~35.7 μM. Only an order of magnitude lower affinity than that measured for the pY1139 peptide with Grb7 SH2 domain [39], it represents a relatively high affinity interaction for a non-phosphorylated peptide. In general the affinity of an SH2 domain/non-phosphorylated peptide interaction is 10^4 ^– 10^5 ^times weaker than the affinity measured for the binding of the SH2 domain to a phosphorylated form of the peptide, with the difference in the total free energy of complex formation largely the result of the electrostatic interactions between the SH2 domain and the phosphate moiety [57,58]. The lack of an electrostatic interaction with a phosphate group may be partially compensated for by the entropic advantage of the cyclized structure of the peptide.

A modeled interaction of the G7-18NATE peptide with the Grb7 SH2 structure, consistent with both the crystallographic and NMR derived data, provides a starting point for understanding the structural basis for the binding specificity observed. As discussed above, the positioning of Tyr 5 against the βD6 (Leu 481) sidechain of Grb7 SH2 may be critical for constructive interactions with the YDN motif. The modeled interaction also demonstrates that the lack of a phosphate moiety on the Tyr5 of G7-18NATE may be partly compensated for by electrostatic interactions between the Glu3 and the positively charged cleft. Whilst glutamate is often used as a pY mimetic, here it may contribute to binding alongside a non-phosphorylated tyrosine. Asp 6 at the +1 position is also likely to be favoured due to its accommodation without steric hindrance in a pocket of the Grb7 SH2 surface with potential for the formation of hydrogen bond contacts. Based on these observations, second generation peptides and peptidmimetic molecules are currently being developed to potently and selectively inhibit Grb7 *in vivo*.

## Conclusion

The crystallographically determined structure and dimer formation of the Grb7 SH2 domain has been described providing a new level of insight into Grb7 target specificity and the likely basis of Grb7 dimer formation *in vivo*. The interaction of Grb7 SH2 domain with a non-phosphorylated inhibitor peptide (G7-18NATE) has been examined, revealing that this peptide binds with unexpectedly high affinity at the classical peptide binding site and that this impacts upon the dimerisation status of the molecule. The structural basis for their interaction has been modeled based on the biophysical data and represents a starting point for the development of second generation inhibitors of Grb7 based cancer cell migration and proliferation.

## Methods

### Protein preparation

The pGex2T plasmid containing the Grb7 SH2 insert (encoding residues 415-532 of human Grb7) was obtained from Dr. Roger Daly [55]. Unlabelled Grb7 SH2 was expressed as a GST fusion protein in *Escherichia coli *strain BL21(DE3).pLysS as previously described [38]. ^15^N-labelled Grb7 SH2 was overexpressed in minimal media containing ^15^NH_4_Cl as its only nitrogen source, using a method similar to that described by Cai and co-workers [59]. Both Grb7 SH2 preparations were purified using glutathione affinity chromatography and cation exchange chromatography following thrombin cleavage of the Grb7 SH2 domain from GST. The molecular mass of the purified unlabelled Grb7 SH2 domain was confirmed using MALDI-TOF mass spectrometry performed by Proteomics International (East Perth, WA, Aus) (M_expected _= 13,672 g/mol, M_observed _= 13,687 g/mol,).

Full-length Grb7 (residues 1 – 532) was subcloned from the mammalian expression vector pRc/CMV into the pGex6P2 expression vector. The identity of the construct was confirmed by DNA sequencing and the construct was used to transform the *E. coli *strain BL21(DE3).pLysS. All cultures were grown in LB containing 100 μg/ml ampicillin and 25 μg/ml chloramphenicol. Full-length Grb7 was expressed as a GST fusion and purified using glutathione affinity chromatography. Full-length Grb7 was removed by on column cleavage with PreScision protease (Amersham) and further purified using cation exchange chromatography. The molecular mass of the purified full-length Grb7 was confirmed using MALDI-TOF mass spectrometry (M_expected _= 60,457 g/mol, M_observed _= 60,415 g/mol).

The purified proteins were dialyzed into 50 mM MES (pH 6.6), 100 mM NaCl and 1 mM DTT, concentrated and stored at 4°C. The final concentrations were determined spectrophotometrically at A_280 _using an extinction coefficient of 8250 M^-1 ^(Grb7 SH2) and 49500 M^-1 ^(full-length Grb7) [60].

### Preparation of G7-18NATE

G7-18NATE (cyclo-(CH_2_CO-WFEGYDNTFPC)-amide) was synthesised manually using Fmoc chemistry and standard solid phase peptide synthesis using RINK resin [61] according to the method described by Pero *et al*. [28]. The peptide was subjected to N-terminal chloroacetylation following the protocol described in Lung *et al*. [62] for the preparation of cyclised G7-18NATE. After cleavage the peptide was cyclised over 48 h in water, with the pH adjusted to > 8 with triethylamine. The cyclised product was lyophilised and purified using reverse phase HPLC and its mass confirmed by mass spectrometry (M_expected _= 1417.6 g/mol, M_measured _= 1417.8 g/mol).

### Crystallization and data collection

The Grb7 SH2 domain was crystallized using the hanging drop vapor diffusion method. 2 μl of protein solution (6.5 mg/ml) was mixed with 1 μl of reservoir solution (100 mM sodium citrate (pH 6.1), 22.5% PEG 4000, 0.2 M ammonium sulphate and 5% glycerol) and incubated over 1 ml of reservoir solution at 22°C. Crystals typically grew within 4 days as square plates with dimensions of 100 × 100 × 30 μm^3^. Crystals were flash-cooled in a nitrogen stream at 100 K and mounted using cryo-loops on a Rigaku RU-H2R rotating anode Cu Kα source (40 kV, 100 mA) equipped with focusing optics and a Mar345dtb image plate detector. X-ray diffraction data were measured to a resolution of 2.1 Å and integrated and scaled with DENZO and SCALEPACK [63]. Structure factor amplitudes were calculated using TRUNCATE [64]. The diffraction data was consistent with the space group *P2*_*1*_*2*_*1*_*2*_*1*_, with unit cell dimensions *a *= 62.6 Å, *b *= 63.9 Å, c = 105.7 Å. The data collection statistics are given in Table 1.

### Structure determination and refinement

Unless otherwise stated all programs used for structural and crystallographic analysis were located within the CCP4 interface [65] to the CCP4 suite [66]. The structure of Grb7 SH2 domain was solved by the molecular replacement method. Initial phases were obtained using MOLREP [67] and the co-ordinates of the Grb10 SH2 domain dimer as the search model (PDB:1NRV, [36]). Attempts to use the NMR model of the Grb7 SH2 domain (PDB:1MW4, [29]) to generate a molecular replacement solution proved unsuccessful. Manual model rebuilding was carried out in O [68] and maximum likelihood refinement was carried out using REFMAC5 [69]. Non-crystallographic symmetry (NCS) restraints were applied between the four chains that occupied the asymmetric unit but were removed for the final round of refinement. Successive rounds of refinement and manual building were performed until peaks no greater than 4σ or less than -4σ were present in a |F_O_| - |F_c_| electron density map. Ordered water molecules were initially added to the model using ARP waters from within REFMAC5 (ARP/wARP version 5.0; [70]) and later were added by hand. Only solvent molecules that were located within good 2F_o _- F_c _electron density and had acceptable hydrogen bonding geometry contacts with either protein or existing solvent were retained in the final model. Sulphate anions were built into regions of tetrahedral shaped electron density. The refinement statistics are reported in Table 1 and the atomic coordinates have been deposited with the Protein data bank. The programs WHATIF [71] and PROCHECK [72] were used to assess the quality of the final structures. Intermolecular contacts were calculated using CONTACT. Buried surface area was calculated using AREAIMOL [73]. The coordinates have been deposited at the RCSB database PDBID: 2QMS.

### Analytical ultracentrifugation

Grb7 SH2 domain and full-length Grb7 were analysed at loading concentrations of A_280 _values of 0.1, 0.3 and 0.6. Sedimentation equilibrium experiments were performed at 20°C in an Optima XL-A analytical ultracentrifuge (Beckman Coulter, Fullerton, CA, USA) at rotor speeds of 14,000, 16,600, 24,300 and 28,800 rpm for Grb7 SH2 and 11,800 and 17,300 rpm for full-length Grb7. Cells fitted with conventional Yphantis 12-mm six-channel equilibrium centrepieces [49] and quartz windows were used. Absorption profiles were recorded at 280 and 360 nm with automatic dialysate absorption compensation. The data were recorded in 0.001-cm steps and ten scans were averaged to produce each profile. The system was deemed to be in equilibrium when profiles recorded 4 h apart were identical. The data were analysed by fitting models of association to the absorbance versus radial position distribution using the program NONLIN [48,49]. The partial specific volumes of Grb7 SH2 domain and full-length Grb 7, calculated from their reported primary sequence, were 0.736 and 0.731 mL/g respectively. The density of the buffer solution was calculated to be 1.00379 g/ml using the program SEDNTERP v1.08 [74].

### Isothermal titration calorimetry (ITC)

ITC experiments were performed on a VP-ITC Microcalorimeter (Microcal, Northampton, MA, USA). Grb7 SH2 domain was dialysed extensively against 50 mM MES (pH 6.6), 100 mM NaCl and 1 mM DTT at 4°C. Lyophilised G7-18NATE was dissolved in the final dialysate and the pH adjusted to 6.6 using 0.1 M NaOH. The reference power was set to 10 μCal/sec and the cell contents were stirred continuously at 310 rpm throughout the titrations. G7-18NATE (440 μM) was titrated into Grb7 SH2 (25 μM) in 30 8 μL injections at 25 °C, with a 5 min delay between each injection, and the heat changes were monitored. A binding isotherm was generated by plotting the heat change evolved per injection against the molar ratio of G7-18NATE to Grb7 SH2. Following baseline correction for heats of dilution (G7-18NATE injected into buffer alone), the binding isotherm was fit by a single binding site model using a non-linear least squares method, using Origin (Microcal Software, Northampton, MA, USA). During fitting the stoichiometry (n) was fixed at 1 while the binding constant (K_d_) and heat of binding (ΔH) were allowed to float.

### NMR spectroscopy: ^15^N-Grb7 SH2 and G7-18NATE HSQC titration experiment

Purified ^15^N-Grb7 SH2 was dialysed against 50 mM sodium acetate, 100 mM NaCl, 5 mM DTT, 1 mM EDTA, 1 mM NaN_3 _pH 6.6 at 4°C and concentrated to 850 μM. Lyophilised G7-18NATE was dissolved in the final dialysate retained from the preparation of ^15^N-Grb7 SH2. Its pH was adjusted to 6.6 using 1 M NaOH and concentration adjusted to 5 mM as determined spectrophotometrically using a molar extinction coefficient of 6970 M^-1^cm^-1^. To each final NMR sample, D_2_O was added to a final concentration of 10 % (v/v).

NMR experiments were performed at 298 K on a Bruker Avance 600 operating at a ^1^H resonance frequency of 600.13 MHz using a 5 mm TXI probe (^1^H, ^13^C and ^15^N) equipped with XYZ-triple gradient capabilities (Bruker). ^1^H,^15^N-HSQC spectra were acquired using adapted versions of the published pulse sequences [75,76]. Water suppression was achieved by replacing the final 90° pulse with a Watergate sequence [77]. The ^15^N-decoupling during acquisition was achieved using the GARP decoupling scheme [78]. The ^1^H carrier frequency was set to that of the water resonance and the ^15^N carrier frequency was set at a frequency between the Arg Nε and backbone amide resonances. Spectral widths of 12.25 ppm (^1^H) and 40.0 ppm (^15^N) were used. Between 32 – 64 scans per increment were collected over 512 *t*_1_increments of 1024 complex data points. The spectra were processed on a Silicon graphics O_2 _workstation using XWINNMR software (Bruker). The ^1^H frequency was referenced to the water signal at 4.80 ppm, calibrated externally using TSP. The digital resolution was enhanced by zero filling to 2048 × 2048 (F_2 _× F_1_) real data points. The spectral resolution of the ^15^N,^1^H-HSQC spectra was enhanced by apodisation with a Lorentz-Gauss function in both dimensions. Baselines corrections were applied by subtraction of an automatically calculated polynomial. Cross-peak volume was calculated using the integration function within the XWINNMR software (Bruker).

### Molecular modeling of the G7-18NATE – Grb7 SH2 complex

The G7-18NATE peptide was constructed using O [68] with the thioether bond generated by defining appropriate topology and parameter files. The peptide was positioned at the surface of the Grb7 SH2 model determined crystallographically by analogy to the interaction between the Shc derived peptide (PSpYVNVQN) interaction with Grb 2 SH2 domain (1JYR; [52]). The structure was subjected to positional refinement using NAMD [79] to ensure there were no steric clashes in the final model.

## Competing interests

The author(s) declares that there are no competing interests.

## Authors' contributions

CJP prepared proteins for biophysical studies, crystallised the Grb7 SH2 domain, built the crystal structure, analysed the AU data, ran and interpreted the NMR experiments and built the protein/peptide model. JMM designed, conducted and analysed the ITC experiment. SEP and JPM designed and conducted the AU experiments. JWS advised and assisted with x-ray data collection and model building. PJL provided initial protein constructs, protocols and expert advise. SCP and DNK are the developers of the G7-18NATE peptide inhibitor of Grb7 and provided expert advise on its preparation and *in vivo *activities. MCJW solved the crystal structure and supervised building and refinement. JAW conceived the study, instructed in protein preparation, peptide synthesis, NMR and AU analysis and wrote the manuscript with CJP. All authors read, contributed to and approved the final manuscript.
